# Cleavage pattern and fate map of the mesentoblast, 4d, in the gastropod *Crepidula*: a hallmark of spiralian development

**DOI:** 10.1186/2041-9139-3-21

**Published:** 2012-09-19

**Authors:** Deirdre C Lyons, Kimberly J Perry, Maryna P Lesoway, Jonathan Q Henry

**Affiliations:** 1 Biology Department, 4115 French Family Science Center, Duke University, Durham, NC 27705, USA; 2 Department of Cell and Developmental Biology, University of Illinois, 601 South Goodwin Avenue, Urbana, IL 61801, USA; 3 Department of Biology, McGill University, 1205 Avenue Docteur, Penfield, Montreal, QC H3A 1B1, Canada; 4 Smithsonian Tropical Research Institute, Apartado Postal 0843-03092, Balboa, Ancon, Republic of Panama

## Abstract

**Background:**

Animals with a spiral cleavage program, such as mollusks and annelids, make up the majority of the superphylum Lophotrochozoa. The great diversity of larval and adult body plans in this group emerges from this highly conserved developmental program. The 4d micromere is one of the most conserved aspects of spiralian development. Unlike the preceding pattern of spiral divisions, cleavages within the 4d teloblastic sublineages are bilateral, representing a critical transition towards constructing the bilaterian body plan. These cells give rise to the visceral mesoderm in virtually all spiralians examined and in many species they also contribute to the endodermal intestine. Hence, the 4d lineage is an ideal one for studying the evolution and diversification of the bipotential endomesodermal germ layer in protostomes at the level of individual cells. Little is known of how division patterns are controlled or how mesodermal and endodermal sublineages diverge in spiralians. Detailed modern fate maps for 4d exist in only a few species of clitellate annelids, specifically in glossiphoniid leeches and the sludge worm *Tubifex*. We investigated the 4d lineage in the gastropod *Crepidula fornicata*, an established model system for spiralian biology, and in a closely related direct-developing species, *C. convexa*.

**Results:**

High-resolution cell lineage tracing techniques were used to study the 4d lineage of *C. fornicata and C. convexa*. We present a new nomenclature to name the progeny of 4d, and report the fate map for the sublineages up through the birth of the first five pairs of teloblast daughter cells (when 28 cells are present in the 4d sublineage), and describe each clone’s behavior during gastrulation and later stages as these undergo differentiation. We identify the precise origin of the intestine, two cells of the larval kidney complex, the larval retractor muscles and the presumptive germ cells, among others. Other tissues that arise later in the 4d lineage include the adult heart, internal foot tissues, and additional muscle and mesenchymal cells derived from later-born progeny of the left and right teloblasts. To test whether other cells can compensate for the loss of these tissues (that is, undergo regulation), specific cells were ablated in *C. fornicata*.

**Conclusions:**

Our results present the first fate map of the 4d micromere sublineages in a mollusk. The fate map reveals that endodermal and mesodermal fates segregate much later than previously thought. We observed little evidence of regulation between sublineages, consistent with a lineage-driven cell specification process. Our results provide a framework for comparisons with other spiralians and lay the groundwork for investigation of the molecular mechanisms of endomesoderm formation, germ line segregation and bilateral differentiation in *Crepidula*.

## Background

Endomesoderm is an ancient cell type that gives rise to a wide array of tissues in bilaterians and its origin and diversification are the subjects of great interest and debate [1-3]. One of the largest assemblages of bilaterians is the Spiralia, which includes mollusks, annelids, sipunculans, echiurans, myzostomids, nemerteans, platyhelminths, entoprocts and gnathostomulids [4]. Despite the profound disparity in their adult body plans, most spiralians share a conserved early cleavage program that allows one to draw direct homology at a single cell level between taxa that have been diverging for hundreds of millions of years. Yet within this conserved framework, variation exists between species, which is documented in the literature going back to the late 19th century [5-10]. This provides unparalleled opportunities for asking how cells, tissue layers and organ systems have evolved within a stereotyped cleavage program [11]. These attributes make spiralians a powerful system for studying the evolution of different tissues, including endomesoderm.

**Figure 1:**
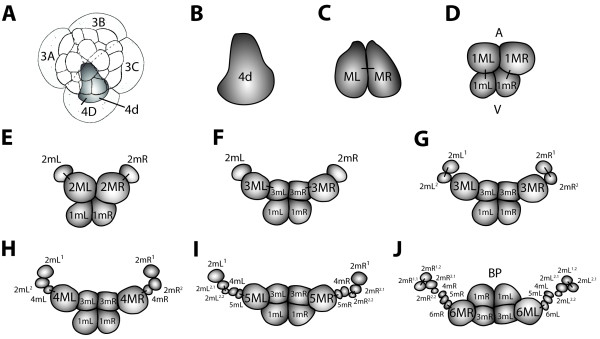
**Diagram showing the cleavage pattern of 4d in *C. fornicata. *(A-B)** Initial formation of 4d at the 25-cell stage and its subsequent cleavage to generate two symmetrical teloblasts, ML and MR. **(C-J)** Subsequent divisions forming the first six pairs of daughter cells, 1mL/R through 6mL/R. The nomenclature used to identify individual progeny is based on that previously proposed by Henry *et al*. [22] and described in the text. The views in **A-C** are animal pole views with the dorsal surface of the embryo located towards the bottom of the illustration. The views in **D-I** are dorsal views. The view in **J** is a ventral view, where the location of the blastopore is shown. Dashes connect sets of sister cells that have just divided. The relative orientation of the animal-vegetal axis is also shown in **D** and applies to parts **D-I**. This relationship is inverted in J as the embryo has been flipped upside-down. Note how the positions and orientation of these cells (bands) change as the embryo undergoes gastrulation and epiboly. A-V: animal –vegetal axis; BP: blastopore.

In many spiralians, mesoderm arises from two distinct regions of the embryo [12]. One source is from the second and third quartet micromeres, and is referred to as ectomesoderm because these quartets also give rise to ectoderm. The other source of mesoderm is a single cell, the 4d micromere, which is referred to as the mesentoblast because it typically contributes to both mesodermal and endodermal fates [10]. The particular micromeres that give rise to ectomesoderm vary among species**,** but 4d gives rise to mesoderm in all embryos that have been studied [10]. In most instances 4d divides bilaterally and these teloblasts generate paired endodermal precursors and mesodermal bands from which definitive visceral mesoderm arises (Figure 1; see [13] as an exception). This behavior represents a critical and profound transition between early spiral cleavage divisions and subsequent bilateral cleavage divisions that help construct the bilaterian body plan in this clade. These characteristics make 4d one of the most conserved features of spiralian development [4]. In some species it is clear that 4d has an additional role as the embryonic organizer that establishes dorsal-ventral polarity [10,14,15]. Although 4d plays an important role in development, there is very little understanding of how this sublineage gives rise to distinct mesodermal and endodermal fates in spiralians. To date, the sublineages of 4d have been investigated using modern lineage tracing in just a few species of glossiphoniid leeches and the sludge worm *Tubifex*[16-19]. Within the mollusks, the early cleavage pattern of the 4d lineage has been reported in the mud snail *Ilyanassa obsoleta*[20,21], but a fate map has yet to be generated.

Here we report a comprehensive fate map of 4d in the slipper snail *Crepidula fornicata* and a closely related species, *C. convexa*. *Crepidula* is a model system for studying the spiralian cleavage program and subsequent development [22,23]. It was in *Crepidula,* in fact, that the term mesentoblast was first applied to the 4d micromere [5,23]. A detailed fate map has already been described for the larval contributions of the first through fourth quartet micromeres and the four fourth quartet macromeres [24]. In that study, 4d was found to give rise to many cell types including the bilateral mesodermal bands, the intestinal hindgut, the left and right velar retractor muscles, and the adult kidney and larval kidney complexes [24]. Henry *et al*. [22] presented preliminary descriptions related to the cleavage pattern of the 4d cell in *C. fornicata* up through the formation of the first four pairs of teloblast daughters (when 12 cells are present in the 4d sublineage). Here we extend and refine that analysis up through the formation of the 6mL/R cells and describe the specific fates of 1mL/R through 5mL/R. We followed the behavior of these 4d sub-clones through gastrulation and early organogenesis stages, and report the ultimate contributions of specific sublineages to preveliger and veliger tissues. We have found that the hindgut comes from the 1m, 3m, 4m and 5m cells, more than were previously thought [23,24]. The 2m cells give rise to many different cell types including cells located beneath the apical organ, muscles surrounding the esophagus, retractor muscles, the absorptive and crystal cells of the larval kidney complex and the presumptive germ cells. Additional tissues, including heart and muscle cells, come from the 6M teloblasts (that is, their later-born daughter cells). We have found that the cleavage pattern and contributions of 4d through the birth of the 4m/4M cells are identical in the direct-developing congeneric species *C. convexa*. We carried out ablations of specific 4d daughter cells in *C. fornicata* and found little evidence of regulation for missing sublineages. These data provide the framework for understanding the mechanistic underpinnings of endomesoderm differentiation, and provide a point of comparison for similar studies in other spiralians.

## Methods

### Collection of adults and embryonic material

Adult *C. fornicata* were collected by the Marine Resources Department of the Marine Biological Laboratory (Woods Hole, MA, USA). Adult *C. convexa* were obtained at low tide from Waquoit Bay near the Waquoit Bay National Estuarine Research Reserve Headquarters (Falmouth, MA, USA). Adults were maintained in sea tables under running natural seawater. Embryos were obtained as previously described [15,22,25,26]. Briefly, embryos were removed from their egg sacs and reared in 0.2-μm-filtered seawater (FSW) at room temperature (approximately 20°C) in gelatin-coated dishes. Penicillin (100 U/mL) and streptomycin sulfate (200 μg/mL) were added to prevent bacterial growth.

### Lineage tracing

**Figure 2:**
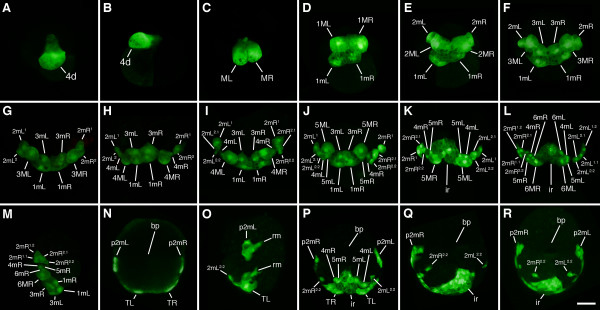
**Confocal fluorescence micrographs showing divisions of 4d in *C. fornicata. *** In each case, the 4d blastomere was injected with rhodamine green dextran (green fluorescence) at the 25-cell stage. Rhodamine green dextran often accumulates within nuclei, which appear to be brighter. Individual cells are as labeled (also refer to Figure 1). **(A,C)** Animal pole views with the dorsal side of the embryo towards the bottom of the figure. **(B)** Right lateral view with the dorsal side of the embryo towards the left. **(D-J)** Dorsal views, with the animal pole towards the top of the figure. **(K**-**L**,**P**-**R)** Ventral (vegetal) views with the right side of the embryo to the left side of the figure. In Q, the view is slightly oblique, with the labeled progeny on the left side of the embryo (right side of this micrograph) not completely visible in this image. **(M)** Right lateral view with the ventral (vegetal) side towards the right side of the figure. **(N)** Dorsal view with the right side of the embryo to the right side of the figure. **(O)** Left lateral view with the ventral (vegetal) side towards the left side of the figure. bp: blastopore; ir: hindgut intestinal rudiment; p2mL and p2mR: clusters of progeny derived from the 2mL and 2mR cells: respectively, which have separated from the teloblast bands; rm: retractor muscle precursors just beginning to leave the 2mL clone; TL: left teloblast; TR: right teloblast (birth number not determined). Scale bar in R equals 50 μm.

**Figure 3:**
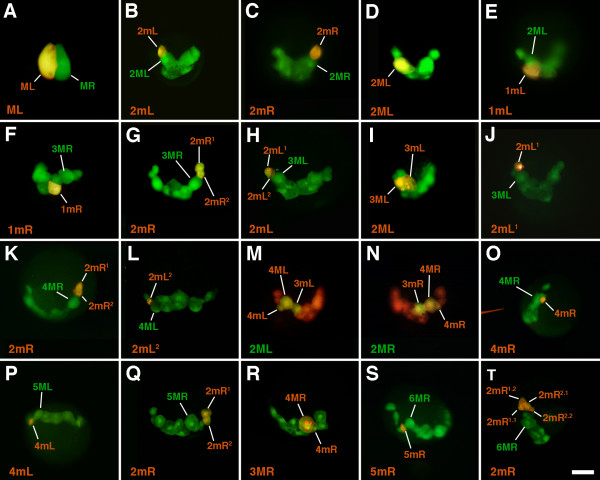
**Photomicrographs showing examples of double-labeled progeny of 4d during early cleavage stages in *C. fornicata. *** In **A-L** and **O-T**, the 4d blastomeres were injected with rhodamine green dextran at the 25-cell stage and selected progeny subsequently injected with DiI (red fluorescence) at a later stage. The DiI-filled injection needle can be seen to the left side in **O**. In **M-N**, 4d was injected with rhodamine dextran and selected progeny subsequently injected with rhodamine green dextran at a later stage. The specific injected progeny of 4d are indicated in the lower left corner of each panel. These cells or their daughters are also labeled in each panel. For orientation, the teloblast is also identified on the side containing the injected cell. Scale bar in T equals 50 μm.

Lineage tracing was carried out as previously described ([22,24]; see also [26]). 4d blastomeres were microinjected at the 25-cell stage with 10,000 molecular weight (MW) rhodamine green dextran (Molecular Probes, Eugene, OR, USA) dissolved in 40% glycerol at a final concentration of 0.2 μg/mL (Figure 2). Cells were injected by pressure injection (Xenoworks digital microinjector, Sutter Instrument, Navato, CA, USA) using glass micropipettes and a fluorescence dissecting microscope. At subsequent stages of development, individual progeny of 4d were then pressure-injected with DiI dissolved in fresh soybean oil (Figure 3; see [22,26-29]). In some cases, 4d was injected with either DiI dissolved in fresh soybean oil or 10,000 MW rhodamine dextran (Molecular Probes, Eugene, OR, USA) dissolved in 40% glycerol at a final concentration of 0.2 μg/mL. Progeny of 4d were subsequently injected with 10,000 MW rhodamine green dextran dissolved in 40% glycerol at a final concentration of 0.2 μg/mL. Cases were examined and photographed live via fluorescence microscopy (see below). Live specimens were mounted as previously described [15]. Image acquisition was done using a Lumenera camera (Lumenera Corp., Ottawa, ON Canada), a Zeiss Axiocam (Carl Zeiss Inc., Munich, Germany), or a Spot Flex camera (SPOT Imaging Solutions, Sterling Heights, Michigan, USA) .

### Fixation and staining

Some embryos and larvae were fixed in 3.7% formaldehyde in FSW for 30 minutes to one hour at room temperature and rinsed in 1X PBS (1.86 mM NaH_2_PO_4_, 8.41 mM Na_2_HPO_4_, 175 mN NaCl, pH 7.4) twice before storage at 4°C. To prevent bacterial growth, 0.01% NaN_3_ was added. Cases were stained with BODIPY FL phallacidin (Molecular Probes, Eugene, OR, USA), which selectively binds the filamentous actin present in differentiated muscle cells. Care was taken to not expose embryos to methanol or ethanol, which is not conducive to phallacidin staining. Specimens were washed three times in 1X PBS and 0.2% Triton X-100 in glass depression dishes. Phallacidin was diluted 1:500 in 1X PBS and 0.2% Triton X-100 by placing the required volume of phallacidin in the empty well of a depression dish, and allowing the methanol diluent to evaporate. The phallacidin was re-eluted in PBS and 0.2% Triton X-100 and specimens were added to this mixture to incubate in the dark at 4°C for 72 to 96 hours. The longer incubation time ensures complete staining of muscles, including those attached deep within the shell of older larvae. Specimens were rinsed three times in 1X PBS and 0.2% Triton X-100 and mounted in 80% glycerin diluted in 1X PBS. Hoechst (1:10,000 dilution) was added to the 80% glycerol in 1X PBS to visualize nuclei.

### Confocal microscopy

Live DiI- and dextran-labeled embryos were mounted in filtered seawater between Rain-X-coated (ITW Global Brands, Houston, TX, USA) glass slides and coverslips supported by clay feet. Embryos were imaged with an inverted LSM 700 confocal microscope (Carl Zeiss Inc., Munich, Germany). Confocal Z-stacks were processed to make maximum projections with ImageJ software (National Institutes of Health, Bethesda, MD, USA).

### Laser ablation

**Figure 4:**
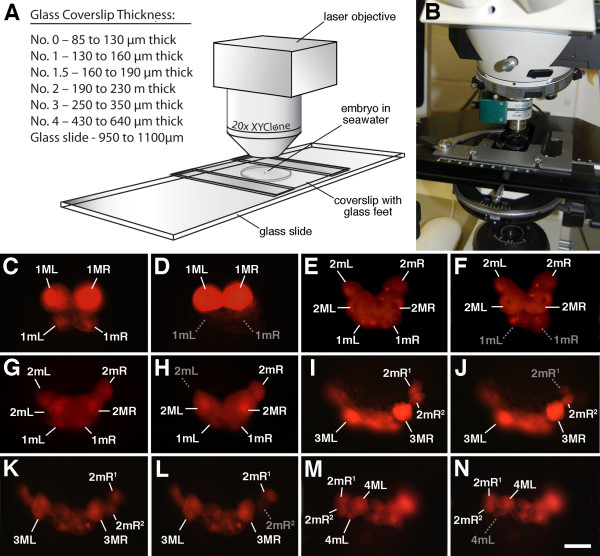
**Use of the XYClone laser for cell ablations in *C. fornicata *embryos. (A)** Diagram showing arrangement of the XYClone laser objective and embryo (small dot) mounted under a raised coverslip supported by glass feet of the appropriate thickness. The approximate thicknesses of commercial numbered coverslips and that of a typical glass slide are provided for reference. See Methods section for explanation on the use of the laser and construction of raised coverslips. **(B)** Photograph showing XYClone laser objective mounted in the upright configuration on a Zeiss compound microscope. **(C**,**D)** Photomicrographs before and after laser ablation of the 1mL and 1mR blastomeres shortly after their birth. **(E**,**F)** Photomicrographs before and after laser ablation of the 1mL and 1mR blastomeres shortly after the birth of the 2mL and 2mR cells. A cloud of faintly fluorescent cell debris can be seen dispersing from the embryo. **(G**,**H)** Photomicrographs before and after laser ablation of the 2mL blastomere shortly after their birth. **(I**,**J)** Photomicrographs before and after laser ablation of the 2mR^1^ blastomere. A cloud of fluorescent debris can be seen dispersing from the embryo. **(K**,**L)** Photomicrographs before and after laser ablation of the 2mR^2^ blastomere. **(M**,**N)** Photomicrographs before and after laser ablation of the 4mL blastomere. A cloud of fluorescent debris can be seen dispersing from the embryo. Gray labels signify the location of missing cells or their dispersing debris. The identities of certain living cells (in white) are shown for reference. Scale bar in N equals 40 μm.

To examine the developmental capacity of selected cells and to look for the necessity of inductive interactions or possible regulation by neighboring cells, specific progeny of 4d were removed using a Zeiss Axioscope equipped with a 20× XYClone laser objective (Hamilton-Thorne, Beverly, MA**,** Figure 4A,B). This objective contains an infrared (IR) laser diode (wavelength approximately 1,450 nm) for precise thermal lysing that destroys individual blastomeres. The embryos are mounted under glass coverslips on Rain-X-coated glass slides. Short, focused pulses of IR energy are delivered to individual cells within the embryos. The IR laser focal waist diameter (to the e^-2^ level) is approximately 4.5 μm, which is collimated to match the visible focal point of the objective. Optimal settings for blastomere ablation need to be determined empirically, and depend on the age and size of the cells. Here each cell (10 to 50 μm in diameter) was ablated using a series of pulses. A train of two to three pulses (set to 100% laser power of approximately 300 mW), each with duration of 200 to 240 μsec delivered over one second, was found to work well without damaging neighboring cells. This was verified by examining the behavior of adjacent labeled cells to see if they continued to divide normally and by examining the resulting cell fates at later stages of development. Laser-ablated cells rupture or swell and are expelled from the embryo in a matter of a few minutes. The loss of the cell and its contents was verified under epifluorescence illumination. The XYClone software calculates and displays the distances for different isotherms of maximum temperature levels, which is useful for helping one to limit collateral damage to adjacent cells. The use of a number of shorter pulses eliminates the potential for such damage due to conductive heating when compared with the use of a single longer pulse. Because the laser beam converges and diverges above and below the focal waist with a conical angle of 26.8°, the beam’s energy becomes spread out above and below the focal point (waist). Combined with the fact that the IR light is highly absorbed by the water (see below), cells above and below the focal point are less likely to be damaged by the beam, and heat energy is rapidly dissipated (conducted) by the surrounding media (that is, sea water). Further discussion of the physics behind the use of IR lasers for the manipulation of eggs and embryonic cells may be found elsewhere [30,31].

The wavelength of 1,450 nm represents the local IR absorption peak for water. Water’s highly efficient absorption of IR light is a property that must be taken into consideration when conducting these experiments. The beam is strongly absorbed by water (absorption coefficient of approximately 28 cm^-1^). The following formula describes the attenuation of IR light as it passes through water:

(1) $$\text{P}\;=\;100\%\cdot \text{exp}\left(-0.0028\cdot \text{X}\right)$$

(2) P=percent power

(3) X=distance in μm

For example, with a distance of 250 μm, IR laser power is reduced to 49.7%, meaning that roughly half of the energy is lost when the light travels through this much water. Therefore, to obtain effective and consistent results, the height of the coverslip must be uniform and the depth of the media kept as shallow as possible. To achieve these conditions and to prevent the embryos from being crushed, the glass coverslips were supported by glass feet of uniform thickness (made from slivers of coverslips of the required thickness, see Figure 4A). These coverslips may be stacked in any configuration to obtain the desired height. The coverslip feet were cut using a diamond scribe and fixed to opposite edges of an intact coverslip using paraffin wax melted on a hot plate. Care must be taken to not use too much wax, which can increase the height of the feet. Any excess wax was removed using a razor blade and the coverslip was then polished clean using tissue paper. In this study we used number 1.5 glass coverslips for both the supporting feet and the top coverslip, which ensured a minimal water path for the laser light to reach the target.

As the XYClone objectives were originally intended for use on inverted microscopes, they are optically corrected for the thickness of plastic culture dishes (approximately 1 mm). To obtain an optimal high-resolution image using an upright microscope, one needs to add a secondary coverslip made from a glass slide (approximately 1m thick, which can also be cut using a diamond scribe). This coverslip can be placed on top of the coverslips described above or used in lieu of a thinner coverslip when affixing the glass feet (see above). The requirement for this secondary coverslip is much less critical when using the 20× XYClone objective, but is essential if one instead uses the 40× XYClone objective, which has a higher numerical aperture.

To visualize the progeny of 4d for laser ablation, the mesentoblast was initially injected with either DiI or rhodamine dextran, which is inherited by all of its progeny. Embryos were viewed via the fluorescence microscope equipped with the laser. Laser target acquisition was done using the computer’s camera display and XYClone software, or the laser system’s virtual ‘heads-up’ ‘RED-I’ spot visible directly through the microscope eyepieces. Live image acquisition for documentation was done using the Luminera camera controlled by the XYClone software (Hamilton-Thorne, Beverly, MA). Other images were captured using a Zeiss Axiocam (Carl Zeiss Inc., Munich, Germany).

## Results

### Overview of *C. fornicata* development

Early development of *C. fornicata* has been described in detail [22-24]. The first two cleavages are essentially equal and parallel to the animal-vegetal axis, establishing four embryonic quadrants, the macromeres A, B, C and D. A small transient vegetal cytoplasmic protuberance called a polar lobe is formed during each of these cleavages and, by the four-cell stage, its contents and plasma membrane are shunted into one macromere, which will become the D or dorsal quadrant [15,23]. Although inheritance of the polar lobe is predictive of which blastomere becomes the D quadrant, its influence on dorsal-ventral axis formation is not completely understood in *Crepidula*. In some species, including *C. fornicata*, polar lobe removal generally has little effect on development or second (dorsoventral) axis formation [15,32]. By contrast, for many other species, the embryo becomes radialized when the polar lobe is removed [33-50]. At the third cleavage each of the four macromeres divides asymmetrically in a dexiotropic direction, giving rise to a quartet of smaller cells at the animal pole called micromeres 1a, 1b, 1c and 1d. The larger, vegetal daughter macromeres are renamed 1A, 1B, 1C and 1D. The macromeres undergo two additional rounds of asymmetric division producing the second and third quartet of micromeres, 2a to 2d and 3a to 3d, in a leotropic and dexiotropic direction, respectively. During this period, the first and second quartet micromeres themselves divide again, so by 18 h past fertilization (hpf) a 24-cell stage is reached consisting of 20 smaller, yolk-deficient micromeres forming an animal cap on top of the four large and yolk-rich 3A-3D macromeres.

**Figure 5:**
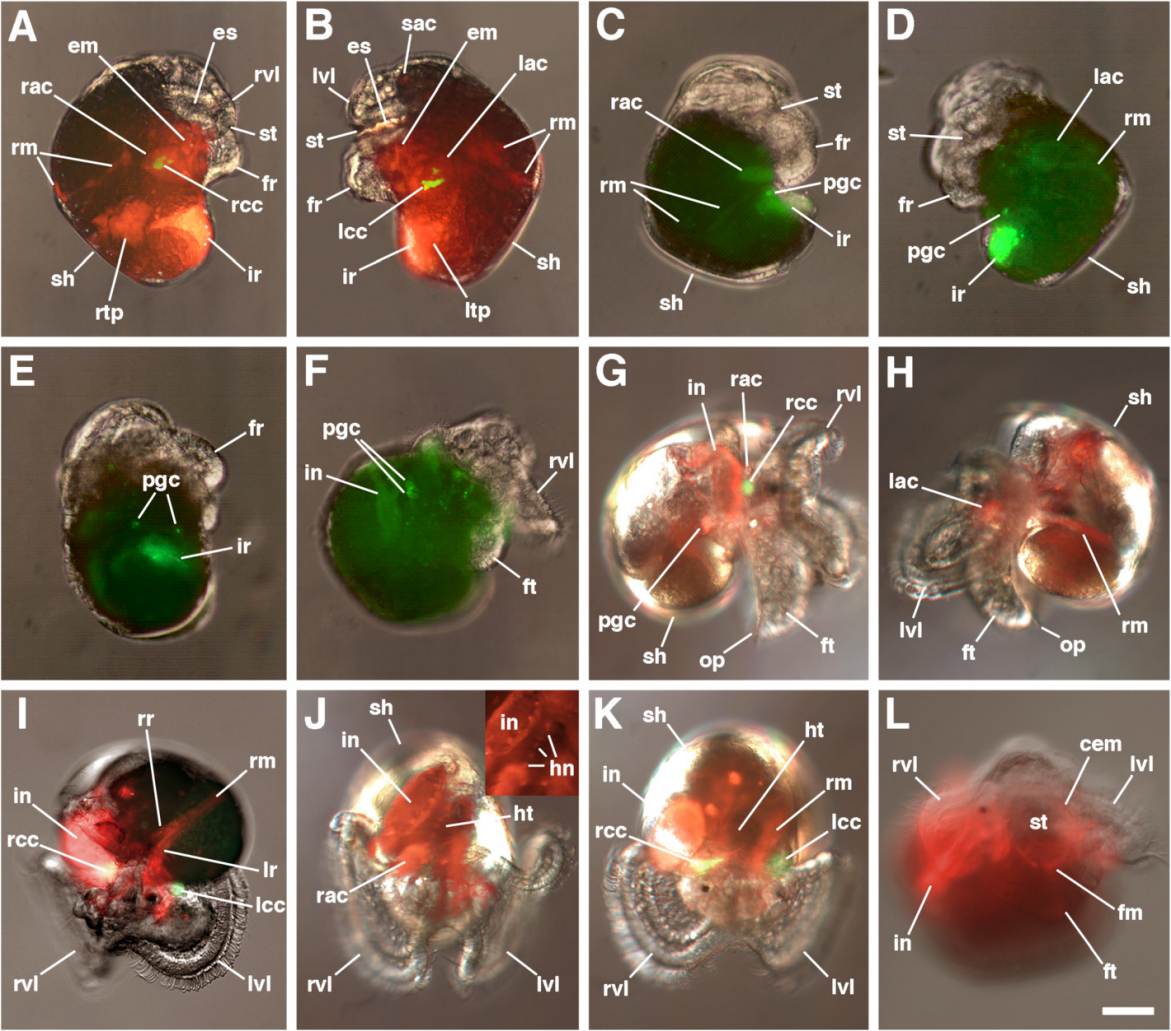
**Progeny of 4d in *C. fornicata. *** In each case, the 4d blastomere was injected with either **(A-B,G-L)** rhodamine dextran (red) or **(C-F)** rhodamine green dextran (green) at the 25-cell stage and allowed to undergo subsequent development. These embryos were raised to various stages of development, as depicted. Photomicrographs reveal the extensive contributions of 4d to different structures within the veliger larva. Green fluorescence in **A-B**,**G**,**I**,**K** show the presence of autofluorescent crystal cells (not included with H, J, L). **A**,**C**,**F**,**G** are right lateral views. **B**,**D**,**H** are left lateral views. E is a posterior, ventral view. I is an oblique frontal view. J,K are superior frontal views. L is an inferior frontal view. cem: circum-esophageal muscles; em: esophageal mesenchyme; es: esophagus; fm: foot muscles; fr: foot rudiment; ft: foot; hr: hindgut intestinal rudiment; hn: heart muscle nuclei; ht: heart; in: hindgut intestine; ir: intestinal rudiment; lac: left absorptive cell; lcc: left autofluorescent crystal cell; lr: left retractor muscle band; ltp: left teloblast progeny; lvl: left velar lobe; mf: muscle fibers; op: operculum; pgc: presumptive germ cells; rac: right absorptive cell; rcc: right autofluorescent crystal cell; rm: retractor muscle; rr: right retractor muscle band; rtp: right telobast progeny; rvl: right velar lobe; sh: shell; sac: sub-apical organ cells; st: stomodeum. Other structures are as labeled in Figure 2. See also text and Figure 6. Scale bar in L equals 40 μm.

**Figure 6:**
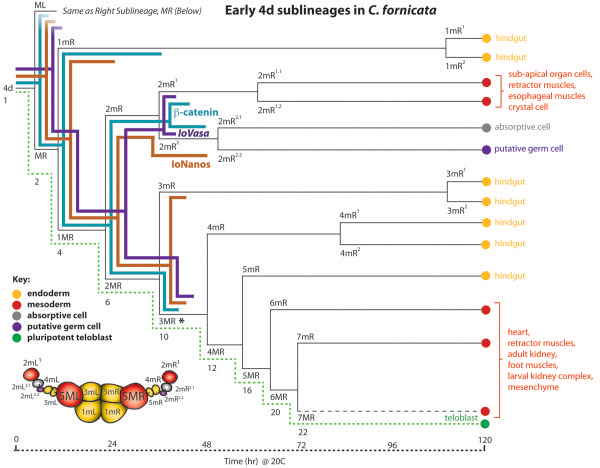
**Cell lineage diagram showing the fates of the first five progeny of 4d’s symmetrical ML and MR teloblasts.** The progeny of the left and right teloblasts ML and MR are equivalent, so only the progeny of MR are illustrated here. A timeline of each set of divisions is also included at the bottom of the illustration. The green dashed line follows the teloblast signified by capital letters (M). Nomenclature follows the convention described in the text and shown in Figure 1 (see also [22]). Fates are color-coded according to their respective germ layers, as indicated in the key. A color-coded diagram is also included showing one representative stage reached when 16 cells are contained within the 4d lineage (stage depicted in Figure 1I). Also included on the fate map are colored lines showing the duration of expression of *IoVasa* mRNA and IoNanos protein as this is detected within specific progeny of 4d, as determined for the closely related caeneogastropod, *Ilyanassa obsoleta* (from [21,51]). In addition, a timeline for expression of green fluorescent protein-tagged beta-catenin in *C. fornicata* is shown (from [22]). *demarks 3ML/R teloblasts, which Swartz *et al*. [51] previously suggested as the progenitors of the germ cells (see also Woods [52,53] for *Sphaerium*).

The fourth quartet of micromeres is born asynchronously. Late in the 24-cell stage, the first sign of overt asymmetry occurs when the 3D macromere protrudes from the embryo in preparation for division. A 25-cell stage is reached by 20 hpf when 3D divides asymmetrically producing the 4d micromere in a leotropic direction (Figure 1A). The division in this quadrant is precocious relative to the others; 3A to 3C will not divide for several more hours (approximately 35 hpf). The 4d micromere inherits some yolk and is larger than the other micromeres. 4d is initially teardrop-shaped with a narrow end extending towards the center of the embryo, which is covered by derivatives of the animal cap, and a larger, rounded part protruding towards the periphery of the embryo, which is exposed (Figures 1A and 2A,B). The 4d cell is the first cell in the embryo to exhibit a bilateral division, giving rise to the paired mesentoblasts (left of the midline (ML) and right of the midline (MR), see Figures 1C and 2C; [23]). These two cells act as stem cells that divide repeatedly to give rise to daughter cells that will contribute to an array of mesodermal and endodermal tissues (Figures 5 and 6; [23,24]).

Gastrulation proceeds over the course of approximately 3 days and during this process the progeny of 4d become fully internalized [22]. Gastrulation begins with the epiboly of the animal cap micromeres to cover the macromeres. By 72 hpf, the embryo also begins to flatten in the animal-vegetal axis so that the equator is the largest circumference of the embryo. Gastrulation is complete by approximately 3.5 to 4 days post fertilization with the remnant of the blastopore prominent on the vegetal side. Organogenesis continues over several days. By approximately 4.5 to 5 days post fertilization, the embryo reaches the preveliger stage when many organ rudiments are distinguishable, including those of the intestine, larval kidney complex, foot, shell gland, velar lobes and stomodeum (Figure 5A-E). The alimentary canal consists of a ventral stomodeum, esophagus, stomach with associated paired midgut glands, style sac, intestine or hindgut, and terminal anus. The external absorptive cells of the larval kidney complex are transient paired lateral structures that protrude laterally from behind the velum and are generally lost by hatching. Each complex contains an external absorptive cell that extends from the surface of the larva, and internal crystal, pore and other supporting cells enclosing the flagellum of the deeper protonephridium [54,55]. The crystal cells harbor green autofluorescent material (Figure 5A-B,G,I,K). The distinctive young veliger stage is reached by 7 to 8 days post fertilization with a prominent shell, large velar lobes, right and left ocelli, foot and operculum (Figure 5F-L). Veligers hatch from the egg case at around 4 weeks and the juvenile stage follows metamorphosis at approximately 6 to 8 weeks [22,23,56,57].

### 4d lineage nomenclature

We employ a new nomenclature for naming 4d progeny in *C. fornicata* (Figure 1), originally introduced by Henry *et al*. [22], as a modification of Conklin’s system [23]. The 4d micromere divides bilaterally to give rise to the paired teloblasts ML and MR located to either side of the midline, where L and R refer to left and right, respectively (Figure 1C). With each successive division, these teloblasts generate subsequent daughter cells, signified by the lower case letter m (for example, 1mL and 1mR; 2mL and 2mR). As each daughter cell is generated, the teloblast is correspondingly numbered in sequence, (for example, 1ML and 1MR; 2ML and 2MR) so that the birth order, age and relationship of these cells can be followed. Superscript numbers (for example, 2mL^1^, 2mL^2^, 2mL^1.1^, 2mL^1.2^) are used in accordance with the standard spiralian nomenclature system [58] for the sequential progeny of the teloblast daughters, as these begin to divide in turn. Daughter cells born closer towards the animal pole or towards the midline are given the superscript 1 and those born closer towards the vegetal pole or more laterally are given the superscript 2.

### Early cleavage pattern of the 4d lineage and behavior of the clone during gastrulation

To follow division patterns in detail, fluorescent lineage tracer was pressure-injected into 4d soon after its birth and embryos were raised to various stages of development and observed live with confocal microscopy (Figure 2). The lineage tracer tends to accumulate in nuclei making it easier to distinguish individual cells, even when they are tightly packed. The slow cell cycles of the teloblast and its daughter cells (Figure 6) made it convenient to study their behavior and provided ample time to inject individual daughter cells within each clone with a second lineage tracer to uniquely label particular sublineages. This allowed one to decipher the order and orientation of the subsequent divisions (examples shown in Figure 3).

**Figure 7:**
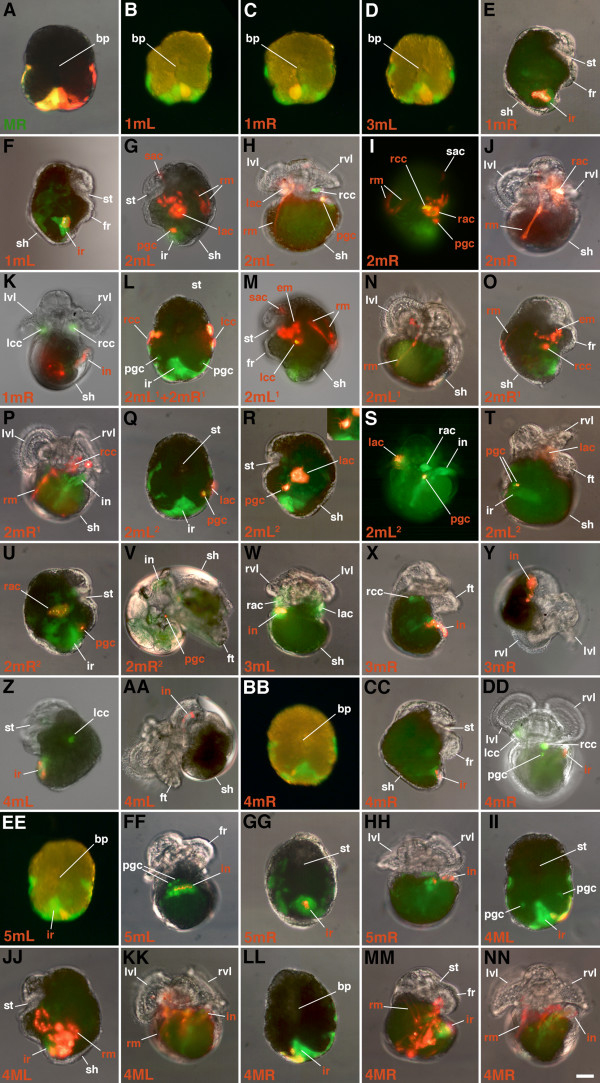
**Differentiated progeny of selected double-labeled daughter cells derived from 4d in *C. fornicata. *(A)** The 4d blastomere was labeled with rhodamine dextran and the MR teloblast was subsequently labeled with rhodamine green dextran. **(B-NN)** The 4d blastomere was injected with rhodamine green dextran (green) at the 25-cell stage and selected progeny subsequently injected with DiI (red) at a later stage. The specific injected progeny of 4d are indicated in the lower left corner of each respective panel. Specific daughters of these double-labeled cells are indicated in each panel. For orientation, the teloblast is also pointed out on the sides containing the injected cells. **A-D**,**L**,**Q**,**BB**,**EE**,**GG**,**II**,**LL** are ventral (vegetal) views with the right side of the larvae to the left side of the figure. Views in **E**,**F**,**I-J**,**O**,**T-V**,**X**,**Y**,**CC**,**MM** are right lateral views. **G**,**M**,**R**,**Z-AA**,**JJ** are left lateral views. **H**,**K**,**N**,**P**,**S**,**DD**,**HH**,**KK**,**NN** are superior views taken from above the head with the right side to the right of the figure. **W,FF** are inferior views taken from below the foot and mouth with the right side of the larvae to the left side of the figure. Inset in R shows higher magnification view of filopodia produced by 2mL^2.2^. Structures are labeled as indicated in Figures 2 and 5. Scale bar in **NN** equals 50 μm.

When 4d is born in *C. fornicata*, it assumes a teardrop shape with the more ventral side of the cell forming a narrow extension into the center of the embryo beneath the animal cap first through third quartet micromeres (Figure 2A,B). 4d’s first division is bilateral (the first to take place in this embryo) and the peripheral, dorsal parts of these cells remain exposed on the surface of the embryo (Figure 2C). As these two teloblasts (ML and MR) divide (Figure 2D,E) and the mesodermal bands get longer, the clone takes on a horseshoe shape with 2mL/R progeny located at its tips towards the anterior/animal poles and the 1mL/R and 3mL/R cells at its center, towards the posterior/vegetal pole (Figure 2F-I). The tips of the mesodermal band, the 2mL/R cells and their progeny lie deep to the micromere cap. Initially 1mL/R and the teloblasts 1ML/R are uncovered, but as the clone expands and the bilateral mesodermal bands elongate, the micromere cap extends over the clone to cover it by the process of epiboly. As this process of gastrulation continues and the ectodermal animal cap advances to surround the embryo and the yolky vegetal macromeres, the embryo becomes flattened along the animal-vegetal axis. The equator assumes the largest diameter of the embryo. As this takes place, the mesendodermal bands now appear to lie along a straightened line extending partway around the equator. Both the 5mL and 5mR cells have been formed by this stage (Figure 2J,K). At even later stages, when one views the embryo from the vegetal/ventral side, the mesodermal bands appear like a horseshoe with arms extending further up the left and right lateral sides of the embryo, towards the anterior pole of the embryo (compare Figure 1I-J; and Figure 2J-L). The configuration of these arms is difficult to view from the dorsal surface. By the time the 6ML/R teloblasts have divided, the progeny of the 2mL/R cells break away from the bands (Figure 2L-O). These cells (initially 2mL^1^/R^1^, 2mL^2.1^/R^2.1^ and 2mL^2.2^/R^2.2^ and later the additional progeny of 2mL^1^/R^1^) remain as the most anterior cells in the bands and become located lateral to the ventral blastopore and future site of the stomodeum (Figures 1J and 2R). Subsequently, the 2mL^2.2^/R^2.2^ cells become relocated towards the ventral midline, lateral and anterior to the cells of the intestinal rudiment (Figure 2O-R). Eventually, cells derived from the 2mL^1^/R^1^ progeny become broadly distributed. For instance, some cells migrate dorsally to form retractor muscles, while two small cells become located at the anterior pole of the larva directly below the apical organ, and still others form dispersed mesenchyme (Figures 5A,B and 7G,KK,MM). The endodermal progeny of 4d remain closer to the vegetal/ventral pole, adjacent to the blastopore, and end up close to and just posterior to the site of the future stomodeum and rudiment of the foot (Figures 5 and 7).

In total, 4d gives rise to a tremendous array of differentiated cell fates, which are illustrated in Figures 5 and 6. These include the hindgut (intestine); the left and right larval retractor muscles; the external absorptive cells and their underlying autofluorescent crystal cells (components of the larval kidney complex); a pair of cells that underlie the apical organ (of unknown function, labeled ‘sac’ in Figures 5 and 7G,I); muscles of the heart, esophagus and foot; widely distributed mesenchyme; and a pair of putative germ cells.

### Contributions of specific 4d progeny and ablation results in *C. fornicata*

To understand the specific contribution of different 4d daughter cells to larval tissues, 4d was injected with rhodamine green dextran and allowed to develop to various stages when one daughter cell was then injected again with DiI (Figure 3). These tandem injected embryos were raised to preveliger and veliger stages to determine the contributions of each cell (Figure 7). For each 4d daughter cell injection, at least 10 cases were examined and similar results were seen for each cell type. In this study, we uniquely labeled the first five teloblast progeny (1mL/R through 5mL/R), and in some instances, their daughter cells (Figure 6). The teloblasts continue to form many additional progeny (6mL/R, 7mL/R, and so on), but the exact number and fates of these cells were not followed in this study. Though generally formed in a synchronous fashion, there may be some slight asynchrony in terms of the relative birth order of the correspondingly numbered left and right daughter cells.

**Figure 8:**
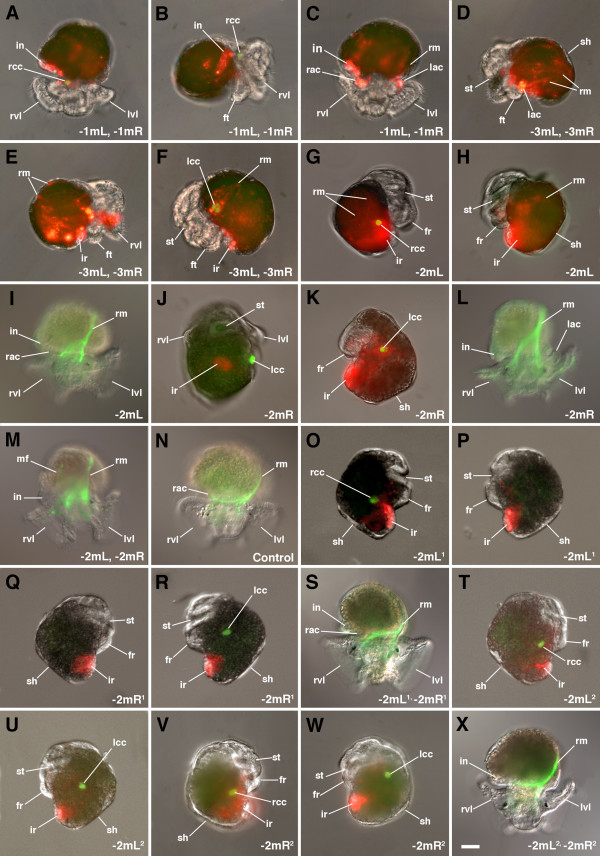
**Examples of embryonic development following laser ablation of specific 4d progeny in embryos of *C. fornicata. *** In each case 4d was injected with DiI at the 25-cell stage and specific progeny ablated at later stages of development. The ablated cell(s) are listed in the lower right corner of each panel. Green fluorescence shows presence or absence of autofluorescent crystal cells. **(A**,**C**,**I**,**L-N**,**S**,**X)** Superior views taken from above the head with the right side of the larvae to the left side of the figure. **(B**,**E**,**G**,**O**,**Q**,**T**,**V)** Right lateral views. **(J)** Ventral view with the right side of the larva to the left side of the figure. **(D,F,H,K,P,R,U,W)** Left lateral views. Structures are as indicated for Figures 2 and 5. Scale bar in X equals 40 μm.

Furthermore, to test for regulation of loss of these cells, each was ablated individually or in matched left and right pairs using an infrared laser (Figures 4 and 8). For each type, at least 10 cases were examined and similar results were seen in each case. Cells were ablated soon after they were born. Cases were discarded if the cleavage was not completed and the sister cell also died (for example, the teloblast).

### Fates of 1mL/R

The first pair of daughter cells formed by the paired ML and MR teloblasts (Figures 1C and 2C) are the 1mL and 1mR cells, respectively (Figures 1D, 2D and 6). The cells are rather large, approximately 50 μm in diameter, and are given off towards the vegetal pole. In general, these cells are formed synchronously, but in some cases the 1mL cell appears slightly larger than that of the 1mR cell. This may be due to the relative packing arrangement and location of these cells. Their sister cells are the teloblasts, and like the initial teloblasts (ML and MR), 1ML and 1MR contact one another along the dorsal midline (Figures 1D and 2D). Injections of fluorescent lineage tracers reveal that both 1mL and 1mR ultimately give rise to proximal and deeper regions of the hindgut endoderm (Figures 2E,F and 7B,C,E,F,K) as has been described previously for *Crepidula*[23] and other mollusks [59]. At the time of their formation, the 1mL and 1mR cells are characterized by the presence of a highly granular, yolky cytoplasm with a vacuolated appearance, previously described by Conklin [23]. This is because there is an animal-vegetal (A-V) gradient of yolk granules within the cytoplasm of 4d and subsequently the two ML and MR teloblasts, with a higher concentration of yolk granules located towards the vegetal pole. At later stages, 1mL/R and their subsequent daughters become elongated along the ventral midline. As the intestinal rudiment begins to form, the proximal region extends to the right side (Figure 7E,F). The clone of cells derived from 1mL lies to the left of and slightly deeper to that derived from 1mR and does not extend all the way to the tip of the extending proximal intestinal rudiment (Figure 7E,F). By contrast, the progeny of the 1mR cell form an elongated spike that extends towards the right side of the embryo during the fifth day of development (see Figure 7E). These cells, along with the progeny of 1mL and additional teloblast daughter cells (see below), are later drawn up the right side of the embryo where they dive deeper to join the visceral endoderm derived from the macromeres [24], as rotation of the visceral mass take place. Throughout this process, the endodermal progeny of 1mL and 1mR remain as a fairly coherent mass of cells.

### Ablation of 1mL and/or 1mR

Removal of either 1mL or 1mR alone does not have a tremendous impact on the appearance of the hindgut, however removal of both cells (Figure 4C-F) leads to certain defects (Figure 8A-C). If the 1mL and 1mR cells are removed, the remaining 3mL and 3mR cells take a position closer to the vegetal blastopore, occupying the former location of the 1mL and 1mR cells during gastrulation. This leaves an apparent void where the 3mL and 3mR cells would normally be situated. The rightward pointed proximal end of the hindgut does not form, and the deeper fluorescing contributions of 1mL and 1mR to the visceral mass appear to be reduced or absent, but the progeny of the remaining 3mL and 3mR cells (along with the 4mL/R and 5mL/R cells) do organize an elongated hindgut tube with an external opening at the anus (Figure 8A-C).

### Fates of 2mL/R

The second pair of daughter cells formed by the paired 1ML and 1MR teloblasts are the 2mL and 2mR cells, respectively (Figures 1E and 2E). Typically these two cells form synchronously though one may form before the other. As noted by Conklin [23], they do not appear as vacuolated as do the 1mL and 1mR cells, described above (Figure 2E). The 2mL and 2mR cells are smaller than the 1mL and 1mR cells, approximately 30 μm in diameter and are formed bilaterally, towards the animal pole (Figures 1E, 2E and 3B,C). As these cells are formed they lie deep beneath the advancing cap of ectoderm derived from the animal micromeres. To inject the 2mL and 2mR cells one must pierce through this overlying ectodermal sheet. These two cells are already present by the time the other fourth quartet micromeres (4a to 4c) are born. At this stage the 1mL and 1mR cells and the teloblasts (1ML and 1MR) still lie in contact with one another along the dorsal midline. The 2mL/R cells and their subsequent progeny make up the tips of the elongating mesodermal bands (Figure 3G,H,J,K,Q,T).

The 2mL/R cells give rise to a large number of progeny (Figures 7G-J and 6). These include progenitors of the left and right bands of the main larval retractor (Figure 7H,J) and specific components of the larval kidney complex that include the external absorptive cells (Figure 7G,I) and the autofluorescent crystal cells (that eventually lie just deep to the absorptive cells; Figure 7G,I). In addition, these give rise to a pair of cells (of unknown function) that become situated beneath the apical organ (labeled ‘sac’ in Figure 7G,I), cells adjacent to the esophagus, and a large quantity of internal mesenchyme. With rotation of the visceral mass the external absorptive cell and underlying crystal cell on the right side become elongated and located at a higher (more anterior) position compared with those on the left (compare Figures 5I and 7H,S,DD).

### Daughters of 2mL/R (2mL^1^/2mR^1^)

As the 2mL/R cells divide, they give rise to 2mL^1^/L^2^ and 2mR^1^/R^2^, respectively (Figures 1G, 2G, 3G,H,J-L,Q,Tand 6). The 2mL^1^/R^1^ daughters are somewhat larger (approximately 25 μm in diameter) and lie more animal and medial relative to their corresponding 2mL^2^/R^2^ sister cells (which are approximately 20 μm in diameter, Figures 1G, 2G, 3G,H,K,L). Subsequently, these cells undergo a series of divisions. The 2mL^2^/R^2^ cells divide first, and the 2mL^1^/R^1^ cells divide several hours later, after the formation of 5mL/R (Figures 1I-J, 2I-L and 6). The more animal 2mL^1.1^/R^1.1^ cells are generally larger than the vegetal 2mL^1.2^/R^1.2^ cells (Figures 1I, 2L,M and 3T). The differential contributions of these latter four cells have not been distinguished. After the birth of 4mL/R the progeny of 2mL/R (2mL^1^, 2mL^2^, and 2mR^1^, 2mR^2^) begin to separate from the other progeny of the teloblasts (Figures 2L-R and 3S,T). It is not clear if this separation is to due active migration or the result of being passively moved by surrounding tissues. Later, as the clones of cells derived from the 2mL/R cells begin to separate away from the other progeny of 4d (by the time that 5mL/R and/or 6mL/R are formed, see below), they are brought to the lateral sides of the embryo and move towards the future anterior pole (Figures 2M and 3Q,T). Initially, these spatially isolated clones contain three and then four cells (2mL^1.1^/R^1.1^, 2mL^1.2^/R^1.2^, 2mL^2.1^/R^2.1^ and 2mL^2.2^/R^2.2^; see Figures 1I-J, 2I and 3T).

The 2mL^1^/2mR^1^ cells give rise to numerous progeny (Figure 7L-P). These cells contribute to the formation of the retractor muscles. The retractor muscle precursor cells move dorsally and join other retractor muscle precursors derived from the 5ML/R teloblasts to give rise to the left and right bands of the retractor muscles. In the process, some of these cells may cross the midline to lie on opposite sides. The 2mL^1^/2mR^1^ cells also give rise to the autofluorescent crystal cells of the larval kidney complex that are lost at the time of hatching. In addition, the 2mL^1^/2mR^1^ cells also give rise to a pair of cells that become situated beneath the apical organ. In 2mL^1^/2mR^1^-injected embryos, cells (including muscles) that surround the esophagus and a large quantity of internal mesenchyme are also labeled.

### Daughters of 2mL/R (2mL^2^/2mR^2^)

In contrast to the 2mL^1^/R^1^ cells, the 2mL^2^/R^2^ cells only undergo a single division, giving rise to 2mL^2.1^/R^2.1^ and 2mL^2.2^/R^2.2^ (Figures 1I and 21). After their birth, these two daughters were never observed to divide again (Figure 6). The 2mL^2.1^/R^2.1^ cells are relatively large cells (Figures 2I and 3T) that give rise to the external absorptive cell of the larval kidney complex (Figures 6 and 7Q-U). These protruding, rounded cells are directly exposed to the capsular environment and are not covered by ectodermal tissue. Formerly referred to as the external larval kidneys, the exact function of these cells is unclear [54,55]. Like the deeper crystal cells derived from 2mL^1^/R^1^, the absorptive cells are lost at the time of hatching.

The more vegetal, 2mL^2.2^/R^2.2^ cells are small (Figures 2I and 3T) and, as mentioned, were never observed to divide again during the larval stage. This is supported by the fact that the fluorescence in these cells remains very bright, presumably from the lack of dilution through subsequent cell division. The behavior and location of 2mL^2.2^/R^2.2^ cells suggests that they represent the primordial germ cells, which we refer to here as presumptive germ cells (Figure 6, see Discussion). During gastrulation, the 2mL^2.2^/R^2.2^ cells become displaced towards the cells of the intestinal rudiment along the ventral midline below the rudiment of the developing foot (Figures 2M,O-R, 5C-G and 7Q-V). It is unclear if they undergo directed active migrations to reach this location. As they migrate they form numerous long filopodia (inset in Figure 7R) and appear to move in an amoeboid fashion towards the cells of the intestinal rudiment (Figure 2O-R). As differential growth and rotation of the visceral mass occurs (separate from the later process of torsion), 2mL^2.2^/R^2.2^ are brought up with the intestinal primordium along the right side of the larva (Figure 7S-U). Eventually these two cells come to lie in contact with one another, next to the intestine and deep near the heart on the right side, adjacent to one of the gut glands (Figures 5F,G and 7V).

### Ablation of 2mL, 2mR and their daughters 2mL^1^/R^1^ and 2mL^2^/R^2^

Removal of either the 2mL and/or 2mR cell leads to the absence of the structures normally formed by these cells, including the autofluorescent crystal cells, the external absorptive cells and putative germ cells (Figures 4G,H and 8G-M). For the most part, these larvae develop with a head and fairly normal external morphology. In cases where both 2mL and 2mR have been removed, the larval retractor muscles are greatly diminished and exhibit somewhat abnormal morphology (Figure 8M). It should be noted, however, that additional retractor muscles are made by other progeny of the teloblast, after 5mL and 5mR have formed, which still contribute to these structures (see below). Cases in which both 2mL^1^ and 2mR^1^ have been removed still have some retractor muscles, which must therefore be derived from additional cells (Figure 8S). However, no formation of the corresponding fluorescent crystal cell(s) is seen. The external absorptive cells formed by 2mL^2^/R^2^ are still present. Removal of 2mL^2^ and/or 2mR^2^ results in the loss of the corresponding absorptive cell(s) and presumptive germ cell(s) (Figure 8M,T-X). Finally, there are beating hearts in both cases in which pairs of 2mL/R, 2mL^1^/R^1^ or 2mL^2^/R^2^ have been removed (see Additional file 1: movie, and data not shown). Thus, any significant contribution of 4d to the development of the heart must be from later progeny of the teloblasts (after 5mL/R have formed). Note that Hejnol *et al*. [24] reported that 2c also contributes to the formation of the heart in *C. fornicata*.

### Fates of 3mL/R

The third pair of daughter cells formed by the paired 2ML and 2MR teloblasts are the 3mL and 3mR cells, respectively (Figures 1F and 2F). The cells are rather large, about 40 μm in diameter, but not quite as large as the 1mL and 1mR daughters. The appearance of 3mL and 3mR is similar to that of 1mL and 1mR, characterized by the presence of a granular, yolky cytoplasm, but not quite as yolky or vacuolated in appearance. 3mL and 3mR are formed from the vegetal and medial sides of the 1ML/R teloblasts and lie in a more animal and superficial location relative to the 1mL/R cells, and in intimate contact with them (Figure 2F). In some cases these cells may lie somewhat more lateral to the 1mL/R cells, and separated from one another (Figure 2G,I). At this stage the teloblasts are no longer in contact with one another as they are separated by the 3mL/R and 1mL/R endodermal precursors. Typically the 3mL/R cells form synchronously, though one can observe one forming before the other. As these cells are formed they lie deep to the advancing cap of ectoderm derived from the animal micromeres that extends via epiboly. Thus, to inject these cells one needs to pierce through the overlying ectodermal sheet. As epiboly takes place the 3mL and 3mR cells may ride up to a slightly more superficial location over the 1mL and 1mR cells.

Like the 1mL/R cells, these cells also do not divide for a few days, around the same time that the 1mL and 1mR cells begin to divide (Figure 6). Injections of fluorescent lineage tracers reveal that both the 3mL and 3mR cells give rise to distal regions of the hindgut endoderm (intestine), as has been described previously [22,23]. Their progeny subsequently generate elongated columns of cells that make up the tube of the hindgut (Figure 7D,W-Y). These progeny extend along most of the length of the intestine. This differs from the behavior of the 1mL/R progeny, which contribute mainly to deeper cells of the digestive tract at the more proximal end of the hindgut tube (Figure 7E,F) and remain as a tighter mass of cells towards the tip of the intestinal rudiment until later in development. The 3mL and 3mR clones appear to undergo the greatest elongation early during gastrulation. These cells extend the furthest to the animal dorsal (posterior) side of the hindgut intestinal rudiment. As the intestine elongates, these progeny become stretched out as discontinuous columns as other progeny (derived from subsequent progeny of the teloblasts, see below) presumably intercalate between these cells. Elongation may be driven by convergent extension and/or directed proliferation. These cells contribute much of the distal length of the hindgut tube that leads all the way to the opening of the anus. The anus begins its formation in a posterior location along the ventral midline (data not shown). As the intestinal tube elongates, the terminal opening (anus) is located in a posterior position below the level of the absorptive cell and to the side of the foot on the right side of the early veliger larva. Eventually the anus will be brought up to a more anterior location above the head when further rotation of the visceral mass and torsion takes place.

### Ablation of 3mL and/or 3mR

Removal of either cell alone does not have a tremendous influence on the appearance of the hindgut, however removal of both cells leads to the absence of an elongated tube and disrupts formation of the anus (Figure 8D-F). The clones of cells derived from the 1mL and 1mR (and other endodermal progenitors, see below) spread out somewhat and do not organize into a coherent mass. Scattered labeled cells can be seen in the embryo. The overall morphology of the larvae appears somewhat abnormal and development is stunted.

### Fates of 4mL/R

The fourth pair of daughters derived from the 3ML and 3MR teloblasts is the 4mL and 4mR cells, respectively (Figures 1H, 2H-M,P, 3M-P,R, and 6). These are very small cells, 10 μm in diameter, formed adjacent to the progeny of the 2mL and 2mR cells (Figures 1H and 2H). They are much smaller than the 2mL and 2mR cells, being approximately the same size or smaller than 2mL^2^ and 2mR^2^. 4mL/R make contributions to the hindgut intestinal tube (Figure 7Z-DD). These cells are typically formed laterally and towards the animal pole off of the 3M teloblasts (Figures 2H,I and 3M-O) and come to lie just vegetal to the progeny of the 2mL/R cells. They may be somewhat shifted laterally or medially relative to the progeny of 2mL/R. Typically these cells form synchronously, though one can observe one forming before the other. The 4mL/R cells typically are born just before the 2mL^2^/R^2^ cells undergo their division to form 2mL^2.1^/R^2.1^ and 2mL^2.2^/R^2.2^ (Figures 1H,I, 2H,I and 6). As these cells are formed they lie deep to the advancing cap of ectoderm derived from the animal micromeres that extends via epiboly. As development progresses, these cells move vegetally (ventrally) relative to the teloblasts; as they join the mass of hindgut endoderm they subsequently pass towards the vegetal tip of the intestinal rudiment derived from the 3mL/R and 1mL/R cells (Figures 2I-M,P and 7Z-CC). After the 4mL/R cells come to lie to the sides of the intestinal mass and have migrated towards the vegetal tip of the hindgut rudiment (adjacent to the blastopore), they begin to divide. By the time the embryo has reached the preveliger stage, they have each formed two progeny (4mL^1^/R^1^ and 4mL^2^/R^2^, Figure 6). Eventually, these cells form an elongated chain of cells along most of the length of the hindgut intestinal tube (Figure 7Z,AA). Progeny of these cells extend all the way to the anus. This behavior is somewhat similar to that seen for the progeny of 3mL/R, as described above. Note that earlier, Henry *et al*. [22] reported incorrectly that 4mL/R contributed to muscles.

### Ablation of 4mL and 4mR

Removal of both 4mL and 4mR leads to abnormal development of the intestine, and the larvae may look somewhat stunted (slowed in development, data not shown). The mass of intestinal cells appears to be smaller in these larvae and the hindgut does not develop normally with the intestinal tube failing to elongate.

### Fates of 5mL/R

The fifth pair of daughters derived from the 4ML and 4MR teloblasts is the 5mL and 5mR cells (Figure 1I). Like the 4mL and 4mR cells, these are small cells, approximately 10 μm in diameter, formed adjacent to the 4mL and 4mR cells (Figures 2J and 3S). In some cases 5mL/R may appear larger than 4mL/R. As these cells form they may lie proximal to the 4mL/R cells or may lie medial to these cells, situated somewhat closer to the vegetal pole (Figure 2J,K). They may be seen to form as part of a coherent band of cells extending from the teloblasts, but their formation may be somewhat variable in location. 5mL/R also contributes to the hindgut intestine (Figure 7EE-HH). These cells end up lying at the posterior base of the intestinal rudiment, to the left and right sides respectively (opposite of the vegetal tip; Figure 7EE,GG). Eventually these cells begin to divide and form a small chain of cells on opposite sides of the terminal hindgut tube (Figure 7FF,HH). Their behavior is somewhat similar to that seen in the progeny of 3mL/R and 4mL/R, as described above (compare with Figure 7W-AA). These cells extend to the opening of the anus as a discrete chain, but do not extend as far as those derived from 3mL/R and 4mL/R. The 5mL and 5mR cells were not removed in this study.

### Later contributions of the teloblasts, 5ML/R

The bilateral teloblasts (for example, 5ML and 5MR) continue to divide and produce a large number of additional progeny (Figures 1I,J, 2J-R, 6 and 7II-NN). The teloblasts continue to generate numerous small daughter cells such as 6mL/R, which form loosely organized chains or clusters (Figures 1J and 2L,M). The 6mL and 6mR cells form next to the 5mL/R and 4mL/R cells. We have not traced the fates of these cells individually in this study. Fates of the later-born teloblast progeny include additional retractor muscles that join those from 2mL^1^/R^1^ (Figure 7II-NN). These fates must also include additional mesenchyme, the heart, the adult kidney and muscles of the foot, as well as other cells that may contribute to the larval kidney complex (for example, protonephridia). Removal of the teloblasts (for example, 4ML/R and 5ML/R) leads to a set of defects including abnormal formation of the retractor muscles, which are not exclusively derived from the 2mL^1^ and 2mR^1^ cells, and loss of a beating heart (data not shown).

### Cleavage and fates of 4d in *C. convexa*

**Figure 9:**
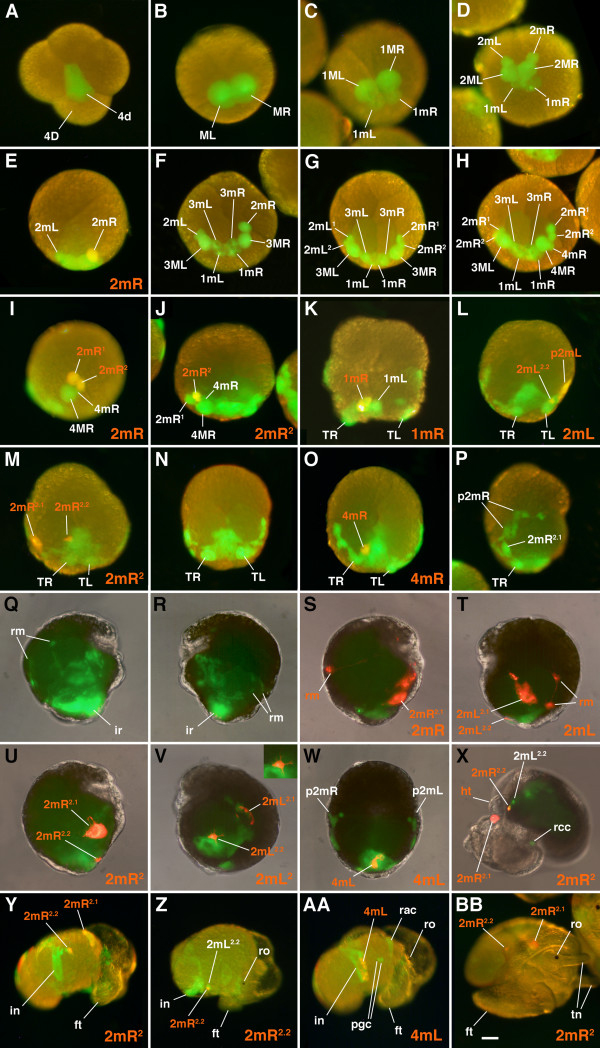
**Cleavage pattern and differentiated progeny of selected daughter cells derived from 4d in the direct-developing embryos of *C. convexa. *** In each case the 4d blastomere was injected with rhodamine green dextran at the 25-cell stage. In some cases (**E**,**I-M**,**O**,**S-BB**), specific progeny of 4d were also double labeled by injection with DiI at a later stage of development. Where appropriate, the specific injected progeny of 4d are indicated in the lower right corner of each respective panel. Specific daughter fates of these double-labeled cells are indicated in each panel. The cleavage pattern and the sublineage fates in the embryos of *C. convexa* are similar to that of *C. fornicata* (compare with Figures 1, 2, 3 and 6). Inset in V shows higher magnification view of filopodia produced by 2mL^2.2^. ro: right ocellus; tn: tentacles. Other structures are labeled as indicated in Figures 2 and 5. Scale bar in **BB** equals 50 μm for **A-X**, 70 μm for **Y-AA**, and 90 μm for **BB**.

In contrast to *C. fornicata*, *C. convexa* is a direct developer. *C. convexa* goes straight to the juvenile stage with very reduced or absent features of larval development [22,60,61]. Cleavage, gastrulation and formation of organs are very similar between *C. fornicata* and *C. convexa* (Figure 9 and see [23]). We followed the progeny of 4d in *C. convexa* through the formation of 4mL/R (Figure 9) and found it to be the same as that in *C. fornicata*. In *C. convexa*, the 4d cell divides giving rise to the bilateral founder cells of the mesodermal bands (Figure 9A-D). The cleavage pattern of the teloblasts is identical to that in *C. fornicata*, as far as we directly observed it up through the formation of 4mL/R and the 4ML/R teloblasts (Figure 9E-H). The divisions within the 2mR/L lineage were also the same; the 2mL/R cells divide to give rise to 2mL^1^/R^1^ and 2mL^2^/R^2^ (Figure 9I). Injections of 2mL/R show label in presumptive retractor muscles, which by analogy should come from the 2mL^1^/R^1^ lineage, and cells in the larval kidney complex and primordial germ cells, which by analogy should come from 2mL^1^/R^1^ and 2mR^2^/L^2^ lineages (Figure 9S,T). Indeed, when the 2mR^2^/L^2^ cells are directly injected (Figure 9M,U,V,X,Y,BB), the absorptive cells are labeled. In addition, a pair of labeled cells are seen to migrate to a position near the prospective gut rudiment made up of the 1mL/R and 3mL/R cells (Figure 9L,M), and later they migrate to a position near the heart, suggesting they are the primordial germ cells (Figure 9Y,BB). When the 2mR^2.2^/L^2.2^ cells are double labeled (Figure 9Z) only the presumptive germ cells contain the dye, and no label is seen in the larval kidney complex, suggesting that it is the 2mL^2.1^/R^2.1^ cells that give rise to the absorptive cell in *C. convexa*, as in. *C. fornicata*. Like *C. fornicata*, the presumptive primordial germs cells in *C. convexa* exhibit amoeboid morphology with prominent filopodial extensions (inset in Figure 9V). When 4mL/R is labeled (Figure 9O), it joins the intestinal rudiment and stretches out along the lumen, as in *C. fornicata* (Figure 9W,AA). We did not carry out ablations for *C. convexa.*

## Discussion

### The 4d lineage in *Crepidula*

The 4d cell is a highly conserved feature of spiralian development as the mother cell of the paired mesodermal bands from which most of the definitive (adult) mesodermal tissue is derived. This role is so highly conserved that there is only one documented case in the polychaete annelid *Capitella* in which 4d does not generate distinct mesodermal bands, though this cell does form mesodermal fates and presumably the primordial germ cells [13]. In many species, 4d also generates endodermal tissues (see Table 1 and [8,10]). In fact Conklin’s original study of *Crepidula* development [23] was the first description of the dual endomesodermal potential of 4d, which had not been described in previous studies for annelids such as *Clepsine*[62] and *Nereis*[58,63]. It is thus important to understand how 4d gives rise to a diversity of tissues and germ layers in order to understand the evolution of endomesoderm in spiralians. The first step is to know the cleavage patterns of 4d and which sublineages give rise to specific tissues. This detailed 4d fate map lays the groundwork for understanding the molecular and mechanistic basis of endomesoderm cell fate and how these tissues are segregated through a stereotyped cleavage program.

**Table 1:** Examples of varied germ layer contributions from the 4d lineage

**Phylum/species**	**Ectoderm**	**Mesoderm**	**Endoderm**	**Primordial germ cells**
**Annelida**				
*Bdellodrilus philadelphicus*	-	+	-	+
[64]				
*Capitella teleta*	+	+	-	+
[13,65,66]				
*Helobdella robusta*	+	+	+	+
[16-18,67-71]				
*Nereis*	-	+	-	N.A.
[58]				
*Platynereis dumerilii*	-	+	-	+
[72-74]				
*Tubifex*	-	+	+	+
[18,19,75]				
**Mollusca**				
*Crepidula fornicata*	+?	+	+	+
[22-24] and here				
*Ilyanassa obsoleta*	-	+	+	NA
[21,59,76]				
*Sphaerium striatinum*	-	+	-	+
[52,53,77,78]				
**Nemertea**				
*Cerebratulus lacteus*	-	+	+	NA
[27]				
**Platyheminthes**				
*Hoploplana inquilina*	-	+	+	NA
[12,79]				

NA: not ascertained; ?: uncertain. References pertain to the studies from which this information was obtained.

### A new nomenclature for 4d

To keep better track of the progeny of 4d, we developed a new nomenclature for the 4d lineage in *Crepidula,* modified from Conklin’s original system (Figures 1 and 6; [22,23]). Conklin referred to the 4d cell as ‘ME’ for ‘MesEntoblast’, and distinguished the left and right progeny with superscripts, ME^1^ and ME^2^, respectively. For subsequent divisions, his original nomenclature referred to mesodermal (M/m) and endodermal (E/e) progenitors with those combinations of letters, respectively. Taking the left, ME^1^ lineage (now ML in our nomenclature, Figure 1) as an example, in Conklin’s system its daughter cells were E^1^ (now 1mL), the primary enteroblast, and Me^1^ (now 1ML), the teloblastic progenitor of mesoderm and endoderm. When Me^1^ divides, it gives rise to m^1^ (now 2mL), the primary mesoblast, and M^1^e^1^ (now 2ML), the mother cell of the mesodermal teloblast and additional endoderm. With the division of M^1^e^1^, Conklin assumed endoderm and mesoderm were fully segregated, and thus the daughter cells were named e^1^ (now 3mL) for the secondary enteroblast and M^1^ (now 3ML) for the mesoblastic teloblast. Hence, Conklin’s nomenclature emphasized the endodermal contributions of 4d, which had been underappreciated or completely overlooked by previous studies, and the emergence of the mesodermal teloblasts ([23], see also [5]).

However, for modern lineage studies, Conklin’s system is cumbersome and impractical for extending the lineage. For example, when m^1^ divides, Conklin names the animal daughter m^1.1^ and the vegetal daughter m^1.2^, in line with the standard nomenclature to distinguish animal versus vegetal daughters in the early embryo [58]. His use of numbers to denote left versus right *and* animal versus vegetal daughter cells is confusing. Instead, we use the postfix L or R to distinguish left and right (for example, ML/MR), and save superscripts for distinguishing the animal and vegetal daughter cells of divisions in non-teloblast lineages (for example, 1mL^1^, 1mL^1.1^; Figure 1).

Another weakness of Conklin’s system is that there is no useful convention for following sequential progeny of the teloblasts or for distinguishing the teloblasts from their daughter cells. Similar weaknesses are present in the system used by Woods [52] where, in fact, two entirely different progeny may carry the same designations. Goulding [20,80] used a modified naming system based on Conklin’s nomenclature that applied the prefix 4d and superscripts for left/right 4d^R^/4d^L^ (equal to ML and MR), which were then followed by numbers to denote the further divisions, with 1 being applied to the more animal daughter and 2 applied to the more vegetal daughter. A specific nomenclature exists for 4d sublineages in clitellate annelids, which develop via a modified spiral cleavage program that gives rise to exceptionally large ectodermal and mesodermal stem cells called teloblasts [9,17,62,81,82]. For example, in the leech *Helobdella* the homolog of 4d, DM" divides bilaterally giving rise to two mesodermal stem cells, the M_L_ and M_R_ teloblasts. Each M teloblast undergoes iterated and highly asymmetric divisions to give rise to a coherent column (bandlet) of primary ‘m’ blast cell cells that each serve as a founder for repeating elements of the segmental mesoderm. The leech has a fixed number of 32 segments, but the M teloblasts produce additional blast cells that also contribute to anterior (early mesoderm or em cells**)** and posterior (supernumerary) nonsegmental tissue. For example, the first six cells born from the M teloblasts give rise exclusively to nonsegmental (em1 to em4), or a mix of nonsegmental and segmental (em5 and em6), tissue. The seventh cell born from the M teloblast (equivalent to 7mL/R in our nomenclature) is the first bona fide segmental founder cell and is called sm1, and subsequent founder cells are numbered in succession [18]. In our system, the teloblast is labeled with a capital letter, and the non-teloblast daughter cell with a lower case letter (for example, 1ML versus 1mL). Additionally, a prefix number is used to keep track of birth order and, with each division, the teloblast is renumbered correspondingly (Figure 1).

Lastly, Conklin’s use of E/e and M/m for endodermal and mesoderm made assumptions about the ultimate fate of these blastomeres, and is limiting. The data presented here clearly show that the separation between endoderm and mesoderm occurs much later in *Crepidula* than Conklin had inferred (Figure 6). In fact, in some species, 4d may not contribute to endoderm formation, such as in *Capitella*[13] (see Table 1). Thus, our naming system builds in no assumptions about ultimate cell fate, and should allow for easier comparisons between different species.

### Contributions to the intestine

In *Crepidula*, the hindgut or intestine is a tube of cells of endodermal origin that contacts the style sac at its proximal (anterior) end and the anus at its distal (posterior) end. Multiple cells born from the teloblasts contribute to the hindgut. The 1mL/R cells give rise to deeper cells of the digestive tract at the proximal end of the hindgut. 3mL/R give rise to the hindgut endoderm, and contribute to most of the distal end of the hindgut tube that leads to the opening of the anus. 4mL/R and 5mL/R also make contributions to the hindgut intestine. There are clearly much more extensive contributions to the formation of the hindgut than previously recognized by Conklin [23], and the separation of endoderm and mesoderm occurs at a later stage of development.

The hindgut is a relatively shorter structure in annelids than in molluscs [83]. In annelids, the hindgut is conventionally considered a proctodeal inturning of ectodermal origin situated posterior to the endodermally-derived midgut, and includes the rectum and anus [84,85]. However, in the polychaete *Capitella*, Meyer *et al*. [13] argue that 4d gives rise to a few cells on the surface of the presumptive anus, which they interpret to be ectodermal (Table 1). In this species, 4d does not give rise to endoderm at all. Conklin reported that cells of the anus or proctodeum are derived from the second and third quartet micromeres in *Crepidula*[23]. He describes that these large cells remain on the surface of the posterior end of the embryo at least through the preveliger stage, that the anus forms much later in development and that the proctodeum is very short. We observed that the 3mL/R through 5mL/R lineages contribute to the most distal end of the intestine leading up to the anus, and these cells are certainly endodermal in origin and clearly not located on the surface of the embryo.

### Contributions to the larval kidney complex

4d contributes to cells of the larval kidney complex, including the superficial external absorptive cell and the underlying crystal cell. The larval kidney complex contains additional deeper cells that include the protonephridia, the pore cell and various support cells (this complex has been well described by [53] and [54]). Notably, the absorptive cells are only present in caenogastropods and are lost at hatching. We have not yet detected whether 4d contributes to the formation of the protonephridia or the adult kidney. One may question whether the absorptive cells, which are exposed on the surface of the larva, represent mesodermal versus ectodermal cells (Table 1). If it is the latter, the 4d cell should be considered an ‘ectomesentoblast’ cell in *Crepidula*.

### Contributions to the heart

The origin of heart tissue in mollusks is controversial, and has been ascribed to the 2c and/or 4d micromeres in different studies [21,23,24,59,77,86-88]. Conklin reported that the second and third quartet micromeres contribute to the larval heart in *Crepidula*[23]. Hejnol *et al*. [24] found that 2c gave rise to both the ectodermal covering and the muscles of the larval heart. Those analyses did not report any contribution of 4d to the heart. In this study, we observe fluorescence in the heart following injection of 4d, which is particularly noticeable in the nuclei of the elongated muscle cells. As the heart beats, it protrudes from the larva between the head and the dorsal edge of the mantle and is covered by a thin layer of ectoderm that appears to be derived from 2c. The heart muscles are very thin elongated cells, and it is difficult to detect label in these cells, which is one reason they might have been missed previously. Furthermore, much of the analysis of Hejnol *et al*. [24] was carried out on fixed specimens and some fluorescence may have been lost.

In *Ilyanassa*, Render [59] reported that the heart was labeled in 2c micromere injections, while removal of the 2c micromere leads to the absence of a beating heart [89]. Yet it has also been reported that removal of the 4d micromere leads to the absence of a beating heart in *Ilyanassa*[59,76,90]. More recently, Rabinowitz *et al*. [21] also observed that the heart was absent when 4d was ablated in *Ilyanassa*. 4d ablations in *Ilyanassa* are particularly informative because, mostly, only 4d-derived tissue is affected, as most (but not all) of the organizing activity of the D quadrant resides in the 3d macromere [21,91]. By contrast, the 4d micromere of *Crepidula* serves as the principle embryonic organizer and when it is ablated the embryo becomes radialized and does not form a heart [15,92]. Consistent with the sublineage fates reported here, we found that sequential removal of paired 1mL/R through 4mL/R daughters does not affect the development of a beating heart, but removal of 4ML/R and 5mL/R do. Additionally, injections of 5mL only label the intestine; therefore, later-born progeny of 4d (derived from 5ML/R) must contribute to this structure.

### Contributions to the germline

It is generally accepted that the germline arises from the 4d lineage in spiralians [13,21,51-53,65,66,72,73,93-96], but exactly when it arises and from what sublineage are not well understood for most species. We argue that the 2mL^2.2^/R^2.2^ cells are the progenitors of the germline in *Crepidula*. This claim is based on several lines of evidence, including comparisons with other model systems, their behavior during gastrulation and their position in the larva. There are claims that the germ cells arise from the pericardial mesoderm such as in the gastropod *Viviparus*[97]. Their close proximity might suggest such an association. However, in *Crepidula* we now know that the heart tissue must arise from a different set of precursor cells than those that give rise to the germline (see above).

Like many germ cell precursors, the 2mL^2.2^/R^2.2^ cells are mitotically quiescent; we never observed either of these two cells dividing up through the veliger stage. The 2mL^2.2^/R^2.2^ cells break away from the mesodermal bands during gastrulation and appear to make amoeboid-like movements while forming dynamic filopodia **(**Figures 2Q,R, 7R and 9V). Their behavior is remarkably similar to the situation encountered for the germ cells of other organisms, including mammals and *Drosophila*[98]. Furthermore, the 2mL^2.2^/R^2.2^ cells take a position in close association, but not necessarily in direct contact with the intestinal rudiment and end up close to the heart. In some other systems, the germ cells migrate along endodermal tissues to reach the gonads [98]. It is possible that the 2mL^2.2^/R^2.2^ cells are following unidentified cues produced by the endodermal cells of the intestine in *Crepidula*.

Another common characteristic of primordial germ cells is a specialized cytoplasmic germ plasm or nuage [99]. In *Crepidula*, there is a distinctive cytoplasm that is associated with the polar lobe that some authors describe as being similar to germ plasm [100-103]. While the polar lobe is known to fuse with the D macromere, it is not known whether the 4d cell inherits the aforementioned specialized cytoplasm. At the eight-cell and later stages the remnants of the polar lobe contents can be detected as a portion of the vegetal cytoplasm - situated adjacent to the vegetal cross-furrow in the D quadrant macromere - that has a different refractive property from the rest of the cell [15]. This cytological feature is not observable directly in the 4d cell. In the course of our present studies we have not observed any unique properties of the cytoplasm of the 2mL^2.2^/R^2.2^ cells that would suggest that they inherit a specialized cytoplasm. Weak and transient *vasa* mRNA staining is seen in 4d in *Crepidula,* but it does not persist into later stages [104]. The localization of Pl10 mRNA has also been studied and no enrichment in the D quadrant or 4d was seen [104]. Other genes in the germline gene regulatory network such as *nanos* and *piwi* have not been examined.

Interestingly, the tips of the bands, including the precursors of the 2mL^2.2^/R^2.2^ cells, preserve beta-catenin protein expression for a long time, suggesting the first evidence of differential molecular signatures among the 4d lineage in *Crepidula*[25]. In the sea urchin embryo, beta-catenin expression persists in the small micromeres as it clears from the rest of the vegetal plate between hatching and archenteron formation [105]. Since the sea urchin small micromeres are known to contribute to the germline [106], this similarity might suggest a conserved role for beta-catenin signaling in germline cell fate.

The origin of the germline within the 4d clone has been examined in a few other spiralians. In *Ilyanassa*, Swartz *et al*. [51] reported that IoVasa mRNA is initially ubiquitous and then becomes restricted to the 4d micromeres and ultimately restricted to the 2mL/R progeny and the 3ML/R teloblasts (Figure 6; cells 4dL^121^ and 4dR^121^ according to their nomenclature), after which staining disappears. Rabinowitz *et al*. [21] also examined IoNanos *in situ* as well as with antibody staining and found that both the mRNA and the protein were initially expressed ubiquitously, then became restricted to the 4d cell. IoNanos protein is mostly associated with the teloblasts, but interestingly it persists longest in the 4dL^112^ and 4dR^112^ cells, equivalent to 2mL^2^/R^2^ cells in our nomenclature (Figure 6). This is suggestive evidence that these cells may represent the germ cell precursors in caenogastropod snails. However, Swartz *et al*. [51] suggested that it is the teloblasts that may represent the progenitors of the germ cells. This assumption was based in part on the earlier studies of Woods [52,53]. In the freshwater bivalve clam *Sphaerium*, Woods [52,53] did a careful study of the emergence of the germline from 4d (which he referred to as d.4 or M). *Sphaerium* possesses a mitochondria-rich and histologically distinct cytoplasm that is present in the egg that segregates to the 4d cell and its two bilaterally symmetrical daughters M and M.1 (equal to ML and MR, respectively). After these teloblasts each form three rounds of mesodermal progenitors, the two remaining teloblasts (called G and G.1, equal to 3ML and 3MR, respectively) each divide equally giving rise to two cells, and these in turn enter a long period of quiescence. Woods [52,53] indicates that these latter four cells are the primordial germ cells, which were followed through late stage development (by virtue of their inheritance of the distinctive population of mitochondria) to eventually appear to join the gonad [53]. Thus, in *Sphaerium*, the germline comes from the equivalent of the 3 M teloblasts.

In *Helobdella* and *Tubifex*, a combination of lineage tracing, ablation and *in situs* for *nanos* and *vasa* have shown that the germline arises from M teloblast-derived segmental mesoderm (sm) blast cell clones contributing to midbody segments [93,95]. Thus, these germ cells are born much later in the 4d lineage relative to those in *Crepidula*, *Ilyanassa* or *Sphaerium*. The germline is known to arise from the 4d lineage in the polychaetes *Capitella*[13,65,66] and *Platynereis*[72], but for the former it is not known when the germline precursors are specifically born. Recently, Rebscher *et al.*[73] showed that, in *Platynereis*, the germline comes from the first two daughters born from the teloblasts (equivalent to 1mL/R and 2mL/R in *Crepidula* by birth rank), which come to reside in the posterior growth zone of the larva.

By all accounts, it appears that the germline arises from 4d in spiralians (Table 1), but the exact sublineage contributions appear to differ between species. In each of these cases, the germline appears to be set aside relatively early during development, and one could argue that it is specified autonomously. Except for *Crepidula*, these embryos exhibit early unequal cleavage divisions and the D quadrant is specified at the four-cell stage. In *Crepidula,* however*,* the early cleavage divisions appear equal and the D quadrant is specified later in development by virtue of inductive interactions from the animal micromeres. There is, however, a bias in terms of which quadrant normally becomes the D quadrant, as only one blastomere (the presumptive D cell) inherits the small polar lobe during the second cleavage division. Whether or not these small polar lobes play a role in germline specification remains to be determined.

### Left/right asymmetries

Render [59] reported some asymmetries for the 4d lineage in *Ilyanassa* in terms of the progeny of the ML (which she referred to as ME1) and MR (which she referred to as ME2) cells. ME1 (ML) contributed to the formation of the larval retractor muscle and the intestine. ME2 (MR) contributed to the formation of the retractor muscle, intestine, the kidney and the heart. Here we have not been able to detect any obvious differences in the fates between the two teloblasts. In fact, the earlier lineages exhibit a great deal of symmetry. It is unclear, however, if there are any later asymmetries that may arise from progeny of the 5ML and 5MR teloblasts after the birth of 5mL/R.

### Induction and regulation within the 4d lineage

We have observed that, in general, when 4d daughter cells are removed, the structures derived from them are missing and little effect is seen in other lineages (Figure 8). However, removal of 3mL/R and/or 4mL/R leads to loss of the elongated hindgut tube, which is derived from 1m, 3m, 4m and 5m cells (Figure 8D-F; note that we did not manage to remove 5mL/R). The retractor muscles are formed mainly by 2m cells and some later-born progeny of the 5M teloblasts. Removal of 2mL and 2mR cells leads to disorganized larval retractor muscles, but they are still visible (Figure 8M). Thus, there could be some inductive interactions controlling the development of these structures as well, but little evidence of any regulation was found to take place. We have not followed 2mL/R^2.2^-ablated embryos to a late enough stage to assess whether definitive germ cells are missing or if other tissues can compensate. The data show that there is very little regulation for missing tissues when specific 4d sublineages are ablated, suggesting that each lineage may be specified autonomously. Elegant cell ablation studies in several clitellate species demonstrated that segmental identity of primary m blast cells (as well as primary blast cells from ectodermal lineages) is specified autonomously at or very near their birth [75,107,108].

In *Ilyanassa*, the 4d cell gives rise to intestine, heart and various other structures [59]. When 4d is ablated these structures are missing, but there are ectodermal defects as well [21]. The shells were smaller and the foot mass was abnormal. Furthermore, Cather [109] suggests that endoderm/archenteron is necessary for the shell gland to develop in *Ilyanassa.* In this species, the 3d macromere carries out a majority of the organizing activity, but the 4d cell has some organizing activity as well, which might explain some of the ectodermal defects in 4d ablations [21]. Since the 4d mesentoblast carries out all organizing activity in *Crepidula*, one might expect to see analogous ectodermal defects when it is ablated. On the contrary, we have not observed dramatic ectodermal defects in our ablations of 4d sublineages, including no obvious reduction of the shell.

### Comparisons of 4d lineage in *C. fornicata* and *C. convexa*

*C. convexa* is a direct-developing species [56,60,61]. The embryos do not form well-developed velar lobes, which are instead vestigial. Unlike in *C. fornicata*, there is accelerated development of the head, foot and the adult shell. Furthermore, we and others have observed that the foot does not form an operculum [22]. This is seen in embryos of large yolky-egged species with direct development. By contrast, species with larval development, such as *C. fornicata,* do form an operculum, as do direct-developers produced from small eggs that increase in size by consumption of nurse eggs (Lesoway, personal observation), but these are subsequently lost at metamorphosis. No adult calyptraeids have an operculum [22]. *C. convexa* does form some transient larval structures, such as components of the larval kidney complex, that include the external absorptive cells and the autofluorescent crystal cells, which are eventually lost at or shortly after hatching. The cleavage pattern of the teloblasts is identical to that in *C. fornicata,* as has been observed through the formation of the 4mL/R and 4ML/R teloblasts (compare Figures 1, 2 and 9).

### Evolution of 4d cleavage patterns in spiralians

One of the enduring paradoxes of studying spiralian development is how such a conserved early cleavage program generates radically different adult body patterns, which may or may not include a hardened exoskeleton (for example, gastropod mollusks versus nemerteans), segmentation (for example, annelids versus nemerteans) and the formation of a coelomic cavity (for example, annelids versus polyclad turbellarians). A crucial intermediate step between the spiral cleavage program and the clade-specific generation of the bilaterally symmetric adult (or larval) body plan is the bilateral division of the 4d cell. Although formation and bilateral division of the 4d cell is often considered one of the most conserved features of spiralian development ([4,10]; see exceptions in [13,79,110,111]), there appears to be a great amount of variation in the cleavage patterns of the 4d daughter cells, the mesodermal teloblasts ([21-23,52,58] and this work). Studying this variation should provide keys to understanding the morphological diversity in spiralians.

In clitellate annelids, DM" (4d) divides making exceptionally large M teloblasts that undergo highly asymmetric divisions and the daughter cells are always born at the same spot off of the teloblast, generating a coherent chain of cells [17,62,81,82]. The 4d cell division pattern in *Crepidula* is very different from this situation. The early cleavages of another caeneogastropod, *Ilyanassa*, are similar overall to *Crepidula*, but exhibit interesting signs of divergence in the arrangement of cells in the clone [20,21]. For example, in both species the first three divisions of the teloblast are reminiscent of spiral cleavage, with the 1m, 2m and 3m cells being born off of alternating sides. Then at its fourth division the teloblast starts to produce a more or less coherent chain of cells. In *Crepidula* this chain extends away from the teloblasts, while in *Ilyanassa* it wraps around the teloblast [21]. The variation in 4d cleavage patterns in mollusks makes an interesting test case for future studies of the evolution of cell division and cell fate within the endomesoderm. For example, in other systems, such as vertebrates and sea urchins, endomesoderm specification happens by inductive signals in a field of cells [105,112], while in spiralians specification is lineage-driven and might rely more heavily on cell autonomous mechanisms such as differential inheritance of cytoplasmic determinants [104,113,114].

### Evolution of novel cell types

Studying spiralian development in a phylogenetic framework has the potential to reveal how novel characters arise within a stereotyped cleavage program. The 4d lineage gives rise to numerous tissues, some of which are almost certainly homologous across spiralian phyla such as the heart, germline and intestine. Other 4d-derived tissues are specific to particular groups such as the retractor muscles in mollusks, the larval kidney complexes in caenogastropods, or the proboscis of glossiphoniid leeches. Clearly, these evolutionarily novel tissues can be readily incorporated into the early division of the 4d lineage, since the founder cells of each arise within the first few divisions of the teloblasts (this work and [18]). Having detailed fate maps for the clonal origin of these tissues is a crucial step to understanding the molecular basis of their evolution.

### Evolution of 4d germ layer contributions

Our data show that, in *Crepidula*, multiple teloblast sublineages give rise to endoderm, while other sublineages give rise to mesodermal fates. Likewise, in *Ilyanassa*, 4d gives rise to both mesoderm and endoderm [21,59]. Although 4d is often referred to as a mesentoblast in the spiralian literature, it does not give rise to endoderm in all species [10,13,18,24]. For example, in the bivalve *Sphaerium*, separation of endoderm and mesoderm happens at the birth of M (4d) and D (4D); 4d gives rise exclusively to mesoderm (and germline) and 4D gives rise exclusively to endoderm (Table 1; [52,53,78]).

Among annelids, 4d’s contribution to the endoderm is more controversial. Several older studies reported no contribution to endoderm (for example, in *Bdellodrilus*[64] and *Nereis*[58], see Table 1). More recently in *Platynereis*, Ackerman *et al.*[74] observed 4d-derived cells as a tight ball at the end of the hindgut, but could not confirm whether these were endodermal cells. These cells might, in fact, be germline precursors [72,73]. The 4d lineage in *Capitella* is particularly unusual because, while it does give rise to the germline, it does not generate discrete mesodermal bands or endoderm [13]. In *Helobdella*, it is traditionally reported that 4d does not contribute to endoderm directly. Rather it does so indirectly when spent M teloblasts and supernumerary m blast cells fuse with the endodermal syncytial yolk cell, which subsequently cellularizes to form the gut epithelium [17,67-69]. However, a recent study found that em1 and em2 (equivalent to 1m and 2m by birth rank) contribute to epithelium that lines the digestive tract directly, and therefore may be considered endodermal cells [18].

## Conclusions

The insights mentioned above emphasize the importance of undertaking careful lineage analyses using long-lived lineage tracers and methods like live imaging using confocal microscopy [13,18,63,115]. A further demonstration is the fact that recent high-resolution lineage studies have revealed that 4d might also produce ectodermal cells. Meyer *et al*. [13] argue that *Capitella*’s 4d cell represents a ‘mesectoblast’ because it gives rise to ectodermal cells that make up the anus (Table 1). In *Helobdella*, 4d appears to contribute to the formation of all three germ layers, because it gives rise to cells in the provisional integument and elements of the nervous system [16,18,71]. Our analysis of *Crepidula* revealed that 4d forms specialized cells exposed on the surface of the larva (the external absorptive cells derived from 2mL^2.1^/R^2.1^[54,55]). If these cells are ectodermal, then the 4d blastomere should be considered an ‘ectomesentoblast’ in *Crepidula* as well (Table 1).

Together, these studies uncover significant diversity in terms of the contributions of 4d in these different spiralian embryos. In reviewing the information summarized in Table 1, which includes a more basal representatives (a polyclad turbellarian in the Platyhelminthes), one might argue that the ancestral condition is one in which 4d gave rise to both endoderm and mesoderm, in addition to the primordial germ cells. Therefore, contributions to ectodermal fates may represent traits more recently derived within certain organisms.

## Competing interests

The authors declare that they have no competing interests.

## Authors’ contributions

All authors contributed to the design and execution of the experiments. DCL and JQH prepared the first draft of this manuscript. All authors contributed to revisions of subsequent drafts. JQH prepared the figures. All authors read and approved the final manuscript.

## Supplementary Material

Additional file 1 **Minus 2mL and 2mR Heart.** This movie depicts a typical veliger larva that forms after removal of both the 2mL and 2mR derivatives of 4d. The larva is depicted as a right lateral view with the shell that surrounds the opaque visceral mass located to the left and the more transparent head and velum to the right. Note the presence of a beating heart towards the top of the larva, which is located between the visceral mass and the head. Click here for file
